# Novel use of superb microvascular imaging in endoscopic ultrasound-guided tissue acquisition for pancreatic mass

**DOI:** 10.1055/a-2515-4160

**Published:** 2025-02-20

**Authors:** Akashi Fujita, Masafumi Mizuide, Shomei Ryozawa, Yuki Tanisaka, Ryuhei Jinushi, Ryuichi Watanabe, Ryo Sato

**Affiliations:** 1 Department of Gastroenterology, Saitama Medical University International Medical Center, Hidaka, Japan

Endoscopic ultrasound-guided tissue acquisition (EUS-TA) is a crucial method for diagnosing pancreatic lesions 1 2 3 4 5 . The Aplio i800 ultrasound device (Canon Medical Systems, Tochigi, Japan) features superb microvascular imaging (SMI), which provides highly sensitive and high-resolution imaging of microvascular flow without contrast agents. SMI has two modes – monochrome SMI (mSMI), which isolates microvascular signals, and color-coded SMI (cSMI), which enhances the visibility of pulsatile flow. These modes complement each other, enabling precise assessment of tumor vascularity and aiding in biopsy site selection, even in challenging cases with heterogeneous or necrotic lesions. This noncontrast imaging technology offers significant advantages in evaluating pancreatic tumors, where accurate visualization of vascularity is critical for diagnosis and treatment planning.

A 73-year-old woman presented with an 83-mm pancreatic body mass identified on computed tomography and suspected pancreatic cancer ( Fig. 1 ). EUS was performed using a linear-array endoscope (UCT260; Olympus, Tokyo, Japan) ( Video 1 ). EUS revealed a hypoechoic, heterogeneous lesion with suspected necrotic areas, making precise biopsy site selection essential ( Fig. 2 ). mSMI provided a detailed, real-time view of microvascular flow, enhancing the understanding of the lesion’s hemodynamics and delineating viable tumor regions ( Fig. 3 ). cSMI offered comprehensive visualization of vascular patterns and the lesion’s overall architecture, facilitating the identification of optimal biopsy targets. Using cSMI guidance, EUS-TA was successfully performed on a viable area of the tumor ( Fig. 4 ). Histopathological examination confirmed adenocarcinoma ( Fig. 5 ). The combined use of mSMI and cSMI ensured detailed and complementary information, leading to a targeted and efficient diagnostic approach.

**Fig. 1:**
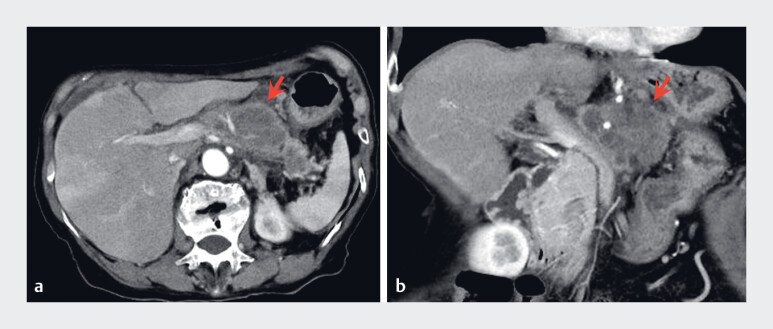
Computed tomography images of the 83-mm mass in the pancreatic body (arrows). **a** Axial view. **b** Coronal view.

By providing detailed imaging of tumor microvascular architecture in real time and without contrast agents, superb microvascular imaging enhanced the accuracy of biopsy site selection, optimized tissue acquisition, and improved diagnostic precision.Video 1

**Fig. 2:**
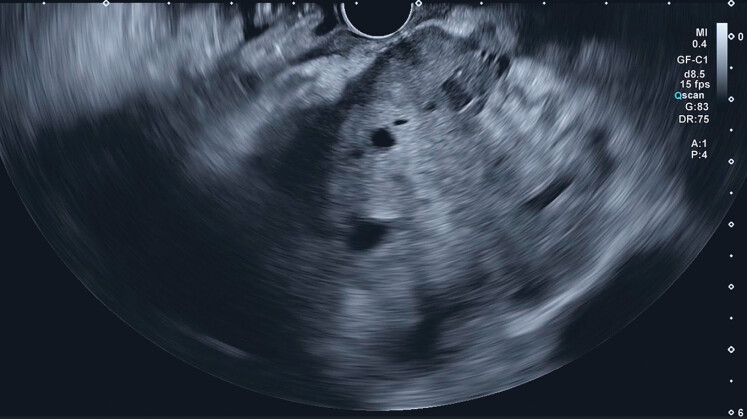
Endoscopic ultrasound B-mode image of the pancreatic body mass (Aplio i800; Canon Medical Systems, Tochigi, Japan), showing a hypoechoic and heterogeneous mass in the pancreatic body, with suspected necrotic areas.

**Fig. 3:**
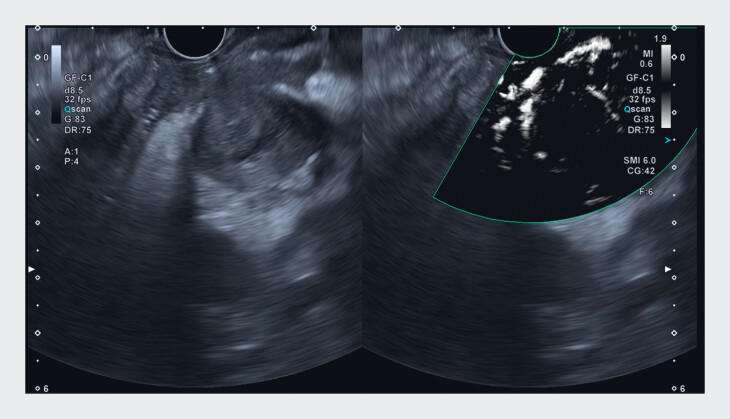
Comparison of B-mode image (left) and monochrome superb microvascular imaging (mSMI) image (right) of the pancreatic body mass (Aplio i800; Canon Medical Systems, Tochigi, Japan). The mSMI image provided a detailed, real-time view of microvascular flow, enhancing the understanding of the lesion’s hemodynamics and delineating viable tumor regions.

**Fig. 4:**
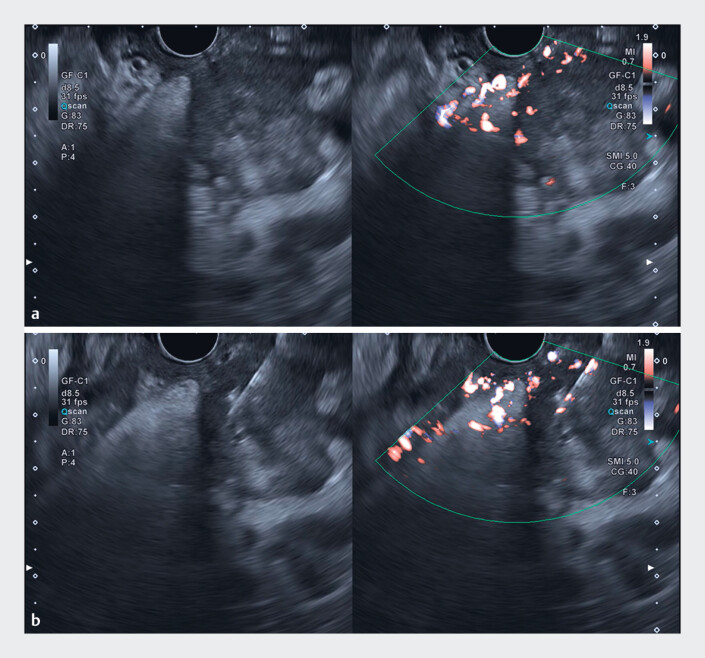
Comparison of B-mode image (left) and color-coded superb microvascular imaging (cSMI) image (right). **a** The cSMI image highlighted the vascular patterns and assisted in delineating viable tumor regions. **b** Endoscopic ultrasound-guided tissue acquisition was performed on a viable region identified with cSMI guidance.

**Fig. 5:**
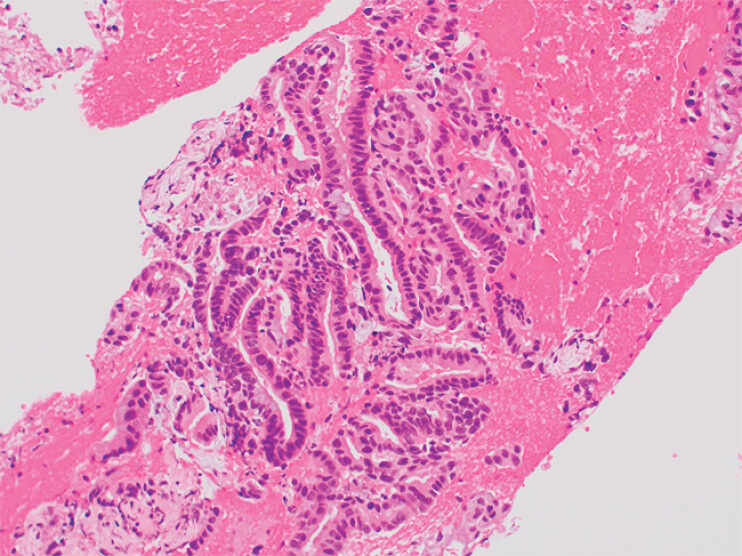
Histopathological findings of the specimen obtained through endoscopic ultrasound-guided tissue acquisition confirmed the diagnosis of adenocarcinoma.

This case highlights the clinical utility of SMI in EUS-TA for pancreatic lesions. By providing detailed imaging of tumor microvascular architecture in real time and without contrast agents, SMI enhanced the accuracy of biopsy site selection and improved diagnostic precision. This innovative technology could be used for other abdominal tumors.

Endoscopy_UCTN_Code_TTT_1AS_2AD

## Notes

Endoscopy E-Videos https://eref.thieme.de/e-videos
*E-Videos* is an open access online section of the journal *Endoscopy* , reporting on interesting cases and new techniques in gastroenterological endoscopy. All papers include a high-quality video and are published with a Creative Commons CC-BY license. Endoscopy E-Videos qualify for HINARI discounts and waivers and eligibility is automatically checked during the submission process. We grant 100% waivers to articles whose corresponding authors are based in Group A countries and 50% waivers to those who are based in Group B countries as classified by Research4Life (see: https://www.research4life.org/access/eligibility/ ). This section has its own submission website at https://mc.manuscriptcentral.com/e-videos .
